# Potential involvement of oxidative stress in cartilage senescence and development of osteoarthritis: oxidative stress induces chondrocyte telomere instability and downregulation of chondrocyte function

**DOI:** 10.1186/ar1499

**Published:** 2005-01-26

**Authors:** Kazuo Yudoh, Nguyen van Trieu, Hiroshi Nakamura, Kayo Hongo-Masuko, Tomohiro Kato, Kusuki Nishioka

**Affiliations:** 1 Department of Bioregulation, Institute of Medical Science, St. Marianna University, Kawasaki City, Japan

**Keywords:** cellular senescence, chondrocyte, osteoarthritis, oxidative stress, telomere

## Abstract

Oxidative stress leads to increased risk for osteoarthritis (OA) but the precise mechanism remains unclear. We undertook this study to clarify the impact of oxidative stress on the progression of OA from the viewpoint of oxygen free radical induced genomic instability, including telomere instability and resulting replicative senescence and dysfunction in human chondrocytes. Human chondrocytes and articular cartilage explants were isolated from knee joints of patients undergoing arthroplastic knee surgery for OA. Oxidative damage and antioxidative capacity in OA cartilage were investigated in donor-matched pairs of intact and degenerated regions of tissue isolated from the same cartilage explants. The results were histologically confirmed by immunohistochemistry for nitrotyrosine, which is considered to be a maker of oxidative damage. Under treatment with reactive oxygen species (ROS; 0.1 μmol/l H_2_O_2_) or an antioxidative agent (ascorbic acid: 100.0 μmol/l), cellular replicative potential, telomere instability and production of glycosaminoglycan (GAG) were assessed in cultured chondrocytes. In tissue cultures of articular cartilage explants, the presence of oxidative damage, chondrocyte telomere length and loss of GAG to the medium were analyzed in the presence or absence of ROS or ascorbic acid. Lower antioxidative capacity and stronger staining of nitrotyrosine were observed in the degenerating regions of OA cartilages as compared with the intact regions from same explants. Immunostaining for nitrotyrosine correlated with the severity of histological changes to OA cartilage, suggesting a correlation between oxidative damage and articular cartilage degeneration. During continuous culture of chondrocytes, telomere length, replicative capacity and GAG production were decreased by treatment with ROS. In contrast, treatment with an antioxidative agent resulted in a tendency to elongate telomere length and replicative lifespan in cultured chondrocytes. In tissue cultures of cartilage explants, nitrotyrosine staining, chondrocyte telomere length and GAG remaining in the cartilage tissue were lower in ROS-treated cartilages than in control groups, whereas the antioxidative agent treated group exhibited a tendency to maintain the chondrocyte telomere length and proteoglycan remaining in the cartilage explants, suggesting that oxidative stress induces chondrocyte telomere instability and catabolic changes in cartilage matrix structure and composition. Our findings clearly show that the presence of oxidative stress induces telomere genomic instability, replicative senescence and dysfunction of chondrocytes in OA cartilage, suggesting that oxidative stress, leading to chondrocyte senescence and cartilage ageing, might be responsible for the development of OA. New efforts to prevent the development and progression of OA may include strategies and interventions aimed at reducing oxidative damage in articular cartilage.

## Introduction

Articular cartilage matrix undergoes substantial structural, molecular, and mechanical changes with ageing, including surface fibrillation, alteration in proteoglycan structure and composition, increased collagen cross-linking, and decreased tensile strength and stiffness [1,2]. Deterioration in chondrocyte function accompanies these changes in the extracellular matrix [3]. Recently, attention has been given to the suggestion that cartilage ageing and chondrocyte senescence play an important role in the pathogenesis and development of osteoarthritis (OA) [4,5]. Several reports revealed that chondrocyte senescence contributes to the risk for cartilage degeneration by decreasing the ability of chondrocytes to maintain and repair the articular cartilage tissue [4-6]. The mitotic and synthetic activity of chondrocytes decline with advancing donor age [5]. In addition, human chondrocytes become less responsive to anabolic mechanical stimuli with ageing and exhibit an age-related decline in response to growth factors such as the anabolic cytokine insulin-like growth factor-I [6]. These findings provide evidence supporting the concept that chondrocyte senescence may be involved in the progression of cartilage degeneration.

Telomeres, the terminal guanine-rich sequences of chromosomes, are structures that function in the stabilization of the chromosome during replication by protecting the chromosome end against exonucleases [7,8]. The telomere DNA may function as a timing mechanism that, when reduced to a critical length, signals a cell to stop dividing and to enter cellular senescence [7-9]. More recent reports demonstrated that the telomere length of chondrocytes shortened with donor ageing and that decreased mean telomere length was closely related to the increase in senescence-associated β-galactosidase expression in human chondrocytes, suggesting that chondrocyte senescence, at least in part, participates in the age-related loss of chondrocyte function responsible for deterioration in articular cartilage structure and function [10]. An understanding of the mechanisms of chondrocyte senescence would be helpful to our efforts to devise new approaches to the prevention and treatment of OA.

Mechanical and chemical stresses are thought to induce increased free radical production, consequently leading to oxidative damage to the tissue [11-14]. Oxidative damage not only can initiate apoptosis through caspase activation but also may lead to irreversible growth arrest, similar to replicative senescence [11,12,15]. Furthermore, it has been reported that oxygen free radicals (O_2_^- ^and peroxynitrite) directly injure the guanine repeats in the telomere DNA, indicating that oxidative stress directly leads to telomere erosion, regardless of cell active division [16]. Generally, it is now thought that oxidative stress/antioxidative capacity may be prominent among factors that control telomere length [17-19]. These findings strongly suggest that oxidative stress could induce chondrocyte telomere instability with no requirement for cell division in articular cartilage, leading to chondrocyte senescence.

Numerous reports have demonstrated that oxidative damage due to the over-production of nitric oxide (NO) and other reactive oxygen species (ROS) may be involved in the pathogenesis of OA [20-23]. However, because of the highly reactive nature of these oxygen reactive species and their short half-lives, it had been difficult to investigate oxidative damage *in vivo *[24]. ROS and NO cannot be directly and accurately measured in a cartilage sample. Recently, a reaction product of ROS and NO, namely nitrotyrosine, was used as evidence of oxidative damage in several ageing tissues [25,26]. Loeser and coworkers [26] demonstrated that nitrotyrosine is over-expressed in normal cartilage from elder donors and in OA cartilage, suggesting the presence of oxidative damage in ageing and degenerative cartilage. These findings provide evidence to support the concept that oxidative stress in articular cartilage affects chondrocyte function, resulting in changes in cartilage homeostasis that are relevant to cartilage ageing, chondrocyte senescence and the development of OA.

Based on the properties of chondrocyte senescence and oxidative stress in OA cartilage, as discussed above, we postulated that oxidative stress induces telomere instability and dysfunction in chondrocytes, subsequently resulting in cartilage ageing and the development of OA through a mechanism involving the acceleration of chondrocyte senescence. It is now thought that oxidative stress/antioxidative capacity is prominent among factors that control telomere length, and hence replicative lifespan [17,18]. To clarify the role of oxidative damage in the pathogenesis of OA, we looked for the presence of oxidative damage in degenerated cartilage from OA patients and examined whether chemical oxidative stress (ROS) affects chondrocyte telomere DNA, replicative lifespan, and function in cultured chondrocytes and in explants of articular cartilage. We also examined the effects of the antioxidative agent ascorbic acid on the oxidative stress induced downregulation of cellular lifespan and function in chondrocytes.

## Methods

### Articular cartilage tissue and chondrocyte culture

Articular cartilage samples were obtained from OA patients (*n *= 9) who had undergone arthoplastic knee surgery (all female, age [mean ± standard deviation] 61.5 ± 5.4 years). The patients had given informed consent, in accordance with the ethical committee of the university. All samples were obtained in accordance with institutional protocol, with review board approval. Donor articular cartilage samples were evaluated macroscopically using a modified Collins scale from 0 to 5, as described previously [27-29].

To obtain sufficient numbers of cells for the experiments, cultured chondrocytes were isolated from macroscopically intact zones of cartilage. Cartilage tissue was cut into small pieces, washed in phosphate-buffered saline (PBS), and digested in Dulbecco's modified Eagle's medium (DMEM; Sigma, St. Louis, MO, USA) containing 1.5 mg/ml collagenase B (Sigma). Digestion was carried out at 37°C overnight on a shaking platform. Cells were centrifuged, washed with PBS, and plated with fresh DMEM.

Basically, chondrocytes were cultured in DMEM supplemented with 10% heat-inactivated foetal calf serum, 2 mmol/l l-glutamine, 25 mmol/l HEPES, and 100 units/ml penicillin and streptomycin at 37°C in a humidified 5% CO_2 _atmosphere [30]. To avoid loss of chondrocyte phenotypes during passages, we used cultured chondrocytes only from passages 1–4. In parallel cultures, we checked the cell morphology and potential to produce proteoglycan in order to examine whether chondrocyte phenotype had been maintained during the passage. Data from chondrocyte mass cultures with loss of chondrocyte phenotypes were excluded from the analysis.

Chondrocytes were cultured in the presence of an antioxidant (100 μmol/l ascorbic acid-2-*O*-phosphate [Asc2P; Wako Junyaku, Tokyo, Japan]) or a ROS (H_2_O_2_) at a concentration of 0.1 μmol/l, which was not cytotoxic to the cells [17]. We had already investigated the effect of H_2_O_2 _(0.1–500.0 μmol/l) on chondrocyte viability *in vitro*. Concentrations of 0.1–200.0 μmol/l of H_2_O_2 _exhibited no inhibitory effects on chondrocyte viability (data not shown). In addition, we had also studied the time course of H_2_O_2 _treatment (0.1–100.0 μmol/l) *in vitro*. Based on our preliminary experiments, in the present study we conducted the cell culture and the organ culture in the presence or absence of H_2_O_2 _(0.1 μmol/l).

In each culture group, the medium including freshly prepared Asc2P or H_2_O_2 _was changed every 2 days. Human chondrocytes were subcultured weekly. At each passage, the total number of collected cells in the dish was determined. Then, 2.5–5.0 × 10^5 ^cells were transferred to a new dish for the next passage, and the number of attached cells was determined 6 hours after seeding. From each passage, the remaining cells after subculture were stored at -180°C until the analysis of cellular activity, telomere length and telomerase activity was conducted.

### Oxidative stress in human articular cartilage

We compared the degree of oxidative stress (antioxidative potential) of the intact cartilage with that of degenerative cartilage tissue. Cartilage samples from the same donor joint were cut and divided into two groups (the degenerated region group, which exhibited macroscopic changes of OA; and the intact region group, which was macroscopically normal).

In these donor matched pairs of articular cartilage samples, antioxidative potential of the tissue was measured using an assay that is based on reduction of Cu^2+ ^to Cu^+ ^and the measurement was conducted according to the manufacturer's instructions (OXIS Health Products, Inc., Portland, OR, USA). This assay measures the total contribution of all antioxidants in the tissue sample. The results of the assay were calculated as mmol/l uric acid equivalents, and expressed as a ratio of antioxidative potential of the degenerating region to that of the corresponding intact region from each donor.

### Immunohistochemistry

For immunostaining of human articular cartilage, paraffin blocks of articular cartilage tissues were prepared using standard histological procedures. Serial sections of paraffin-embedded bone and cartilage tissues were cut and immunostained using an antibody for nitrotyrosine. The sections were deparaffinized and hydrated. Then, the slides were stained using horseradish peroxidase method [26]. Briefly, the slides were blocked with 3% H_2_O_2_. After blocking nonspecific protein binding with blocking agent (Dako, Carpinteria, CA, USA), the sections were incubated with a monoclonal antibody to nitrotyrosine (1:100 dilution; BIOMOL Research Laboratories Inc., Plymouth Meeting, PA, USA) for 1 hour at room temperature, followed by incubation with biotinylated goat anti-mouse IgG (Dako) for 30 min at room temperature. After washing with PBS, the sections were incubated with streptavidin–horseradish peroxidase complex (LSAB2 kit; Dako) for 30 min at room temperature We used diaminobenzidine (Sigma) as a visible peroxidase reaction product. Sections were counterstained with Mayer's haematoxylin (Sigma).

Cells positive and negative for nitrotyrosine were counted in the 20 areas of cartilage at 200× magnification (0.785 mm^2^/field). The level of immunostaining for nitrotyrosine was expressed as a mean number of nitrotyrosine-positive cells per field.

### Chondrocyte activity

Chondrocyte activity was measured as the production of glycosaminoglycan (GAG) by cultured chondrocytes [15]. After undergoing continuous treatment with ROS or ascorbic acid (initial subculture at the start of the experiment: 1 × 10^5 ^cells/dish, chondrocytes from passage 2), the cells were collected with trypsin and washed with PBS. Then, chondrocytes (1 × 10^5 ^cells/dish) were plated in the culture dishes and incubated for 12 hours, and the amount of GAG in the supernatant was measured using a spectrophotometric assay with dimethylmethylene blue (Aldrich Chemical, Milwaukee, WI, USA) [31].

### Determination of the lifespan of cultured chondrocytes

The increase in cumulative population doublings at each subculture was calculated based on the number of cells attached and the cell yield at the time of the next subculture. Population zero was the primary culture of human chondrocytes, and the number of each successive generation was calculated using the following formula [32,33]: generation number at the start of the subculture + log_2_([the number of collected cells at the time of the next subculture]/[the number of attached cells at the start of the subculture]). Senescence was defined as less than one population doubling in 4 weeks. The *in vitro *lifespan (remaining replicative capacity) was expressed as population doublings up to cellular senescence [34].

### Telomere length of cultured chondrocytes

Telomere length was determined using terminal restriction fragment Southern blot analysis, as described previously [35,36]. Genomic DNA from 10^6 ^chondrocytes from each subculture (initial subculture at the start of the experiment: 1 × 10^6 ^cells/dish, chondrocytes from passage 3 or 4) was digested with 400 μl DNA extraction buffer (100 mmol/l NaCl, 40 mmol/l Tris [pH 8.0], 20 mmol/l EDTA, and 0.5% SDS) and proteinase K (0.1 mg/ml). Extraction was performed using phenol chloroform. Extracted DNA (5–10 μg) was digested with 10 units of *Msp*I and *Rsa*I (Boehringer Mannheim, Indianapolis, IN, USA) for 12–24 hours at 37°C. The integrity of the DNA before digestion and the completeness of digestion were monitored by gel electrophoresis. Electrophoresis of digested genomic DNA was performed in 0.5% agarose gels in 45 mmol/l Tris-borate EDTA buffer (pH 8.0) for a total of 660–700 V-h. After electrophoresis, gels were depurinated in 0.2 N HCl, denatured in 0.5 mol/l NaOH and 1.5 mol/l NaCl, transferred to a nylon membrane using 20× SSC, and dried for 1 hour at 70°C. The telomeric probe (TTAGGG)_3 _(Genset, La Jolla, CA, USA) was 5' end-labelled with [α-^32^P]ATP using T4 PNK (Boehringer Mannheim). Prehybridization and hybridization were performed at 50°C using 5× Denhardt's, which was composed of 5× SSC, 0.1 mol/l Na_2_HPO_4_, 0.01 mol/l Na_4_P_2_O_7_, 30 μg/ml salmon sperm DNA, and 0.1 mmol/l ATP. The mean terminal restriction fragment length was determined from densitometric analysis of autoradiograms, as described previously [35].

### Tissue culture of human articular cartilage

Procedures for preparing articular cartilage were generally the same as mentioned above. Briefly, articular cartilage was excised in small, full-depth slices (typically 1.0 cm square) from patients with OA (*n *= 4) who had undergone arthroplastic knee surgery (all females; ages 61, 65, 67 and 68 years). The cartilage explants were cut, weighed and divided into three groups as follows: control group, antioxidative agent + oxidative stress treated group, and oxidative stress treated group. Control and experimental cartilage explants (site-matched pairs) were placed in individual dishes (diameter 6.0 cm) with 10.0 ml DMEM with 10% foetal bovine serum, 100 units/ml penicillin/streptomycin. The process of harvesting the cartilage tissue resulted in significant catabolic activity that was measurable in the absence of interleukin-1 stimulation, presumably due to secretion of proteases in response to trauma. The contribution of this basal catabolic activity could be minimized by culturing for 24 hours before aspiration of the culture medium, washing with PBS, and adding fresh culture medium [37,38]. For the antioxidative agent + oxidative stress treated group, the cartilage explants were incubated in the culture medium with 100.0 μmol/l Asc2P plus 0.1 μmol/l H_2_O_2_. For the oxidative stress treated group, the explants were incubated in the culture medium in the presence of 0.1 μmol/l H_2_O_2_. For each group, culture medium including freshly prepared Asc2P or H_2_O_2 _was changed every day.

At the end of each incubation period (48, 72, 96, 120 and 120 hours), the cartilage samples and the culture media were collected and re-weighed for analyses. The cartilage samples were washed with PBS. Some parts of cartilage samples were fixed with 4% paraformaldehyde at 4°C, and then paraffin blocks were prepared using standard histological procedures. For nitrotyrosine staining, the sections were deparaffinized and hydrated, and then were immunostained using antibody for nitrotyrosine in accordance with the method described above.

Other cartilage samples and supernatants were stored at -80°C for the determination of GAG concentration and isolated chondrocyte telomere length. Catabolic changes to GAG in cartilage were analyzed by determining the GAG content remaining in cartilage tissue relative to the total amount of GAG in the culture (GAG released into the culture media plus GAG in the tissue) in the presence of the antioxidative agent or ROS [2,39]. GAG contents were measured using a spectrophotometric assay mentioned above. Procedures for cultured chondrocyte preparation from tissue cultured explants and telomere length assay were generally the same as those described above.

### Statistical analysis

Results were expressed as a mean value ± standard deviation. Comparison of the means was performed by analysis of variance. *P *< 0.05 was considered statistically significant.

## Results

### Oxidative damage in human articular cartilage tissues

To determine whether oxidative damage was present in OA degenerated cartilage, we measured the antioxidative potential of the intact region and degenerated region isolated from the same articular cartilage tissue of patients who had undergone arthroplastic knee surgery. In the donor-matched pair of intact and degenerated regions from same articular cartilage, the antioxidative potential in the intact region was significantly greater than that in the degenerated region of articular cartilage in the OA patient group (*n *= 9; mean percentage antioxidative capacity of degenerative cartilage compared with intact cartilage: 45.5 ± 16.8%), suggesting that degenerated cartilage may exhibit more oxidative damage than an intact region from the same OA cartilage.

### Presence of nitrotyrosine in articular cartilage from patients with osteoarthritis

To clarify the relationship between oxidative damage and development of OA, immunostaining for nitrotyrosine was examined in the donor-matched pair of intact and degenerated articular cartilage sections from the same OA sample.

**Figure 1:**
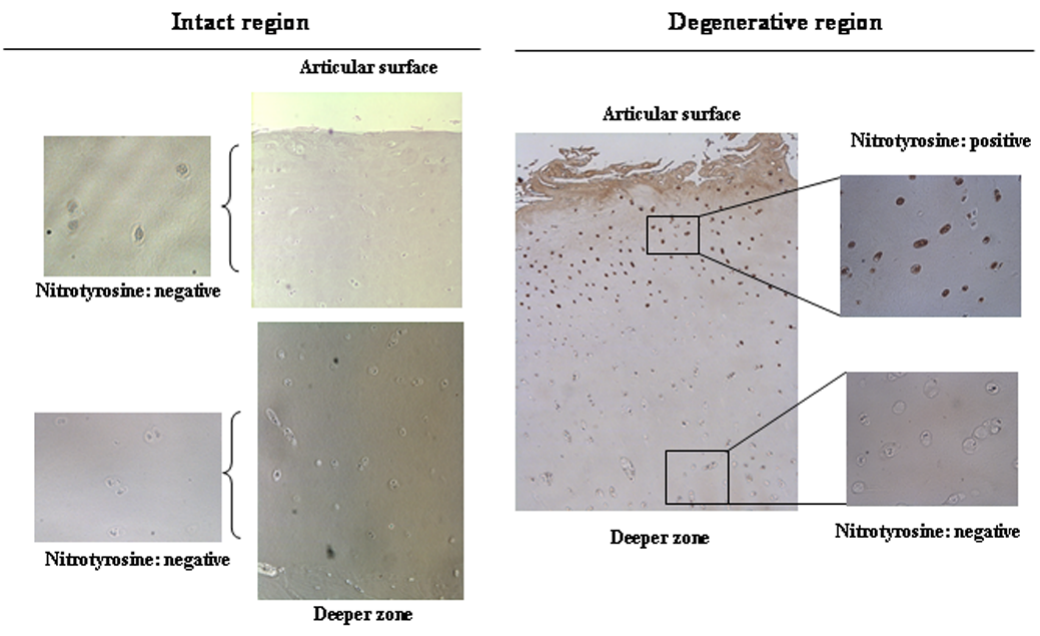
Representative immunohistochemical staining for nitrotyrosine in donor articular cartilage. Cartilage sections were immunostained using an anti-nitrotyrosine antibody. In donor-matched pairs of degenerative and intact regions from same cartilage explants (67-year-old donor), positive immunostaining for nitrotyrosine was observed in chondrocytes and in the cartilage matrix in degenerated regions, whereas the intact region from same cartilage sample showed no positive staining for nitrotyrosine. Original magnification: 40×.

Figure 1 shows a representative example of immunohistochemical staining for nitrotyrosine in the articular cartilage from an OA patient (female, 67 years old). Immunostaining for nitrotyrosine was most apparent in the degenerated regions of articular cartilage that showed histological changes consistent with OA (nine patients; positive cells/field, intact cartilage versus degenerated cartilage: 0.3 ± 0.1 versus 7.4 ± 2.4; *P *< 0.01). Nine of 10 donor samples with degenerated regions were highly positive for nitrotyrosine. Nitrotyrosine was present both within chondrocytes and in the cartilage matrix, and was seen mainly in the more superficial regions. The degree of immunostaining for nitrotyrosine (number of positive cells/field) correlated with the level of histological change in donor cartilage tissues (*n *= 9, r^2 ^= 0.4671; *P *< 0.01). In contrast to the immunostaining in the degenerated regions, almost all intact regions isolated from the same articular cartilage were negative for nitrotyrosine, even in superficial and deep zones (Fig. 1).

### *In vitro *chondrocyte activity under the different oxidative conditions

**Figure 2:**
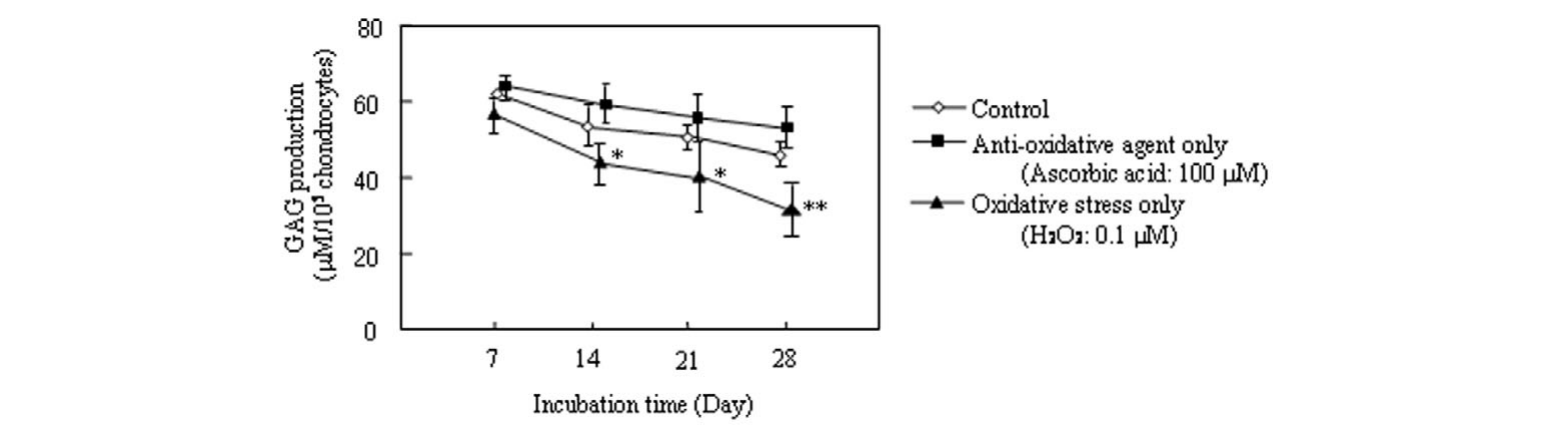
Glycosaminoglycan (GAG) production from cultured chondrocytes under different oxidative conditions. After the incubation times indicated, in the presence of 0.1 μmol/l H_2_O_2 _or 100.0 μmol/l ascorbic acid (initial subculture at the start of the experiment: 1 × 10^5 ^cells/dish, chondrocytes at passage 2), chondrocytes were collected and transferred to a new culture dish (1 × 10^5 ^cells/dish). Following 12 hours of incubation, the amount of GAG in the supernatant was measured using a spectrophotometric assay with dimethylmethylene blue. Values are expressed as the mean ± standard deviation of nine donors (*n *= 4 culture dishes per treatment group at each incubation period; *P < 0.05, **P < 0.01, versus control group at each incubation time). The H_2_O_2 _treated group exhibited a significant decrease in GAG production by chondrocytes as compared with the control group at all incubation times. In the antioxidative agent group the level of proteoglycan production tended to increase as compared with the control group, although no significant differences were observed between the control groups and antioxidative agent groups at any incubation time.

Figure 2 shows that GAG synthesis from cultured chondrocytes decreased gradually in a time dependent manner, regardless of the presence of H_2_O_2 _or an antioxidative agent *in vitro*. The H_2_O_2 _treated group showed a significant decrease in proteoglycan production by chondrocytes as compared with the control group at any incubation time. In contrast, in the antioxidative agent group the level of proteoglycan production tended to increase as compared with that in control groups, although no significant differences were observed between control groups and antioxidative agent groups at any incubation time (Fig. 2).

### Chondrocyte replicative potential under the different oxidative conditions

**Figure 3:**
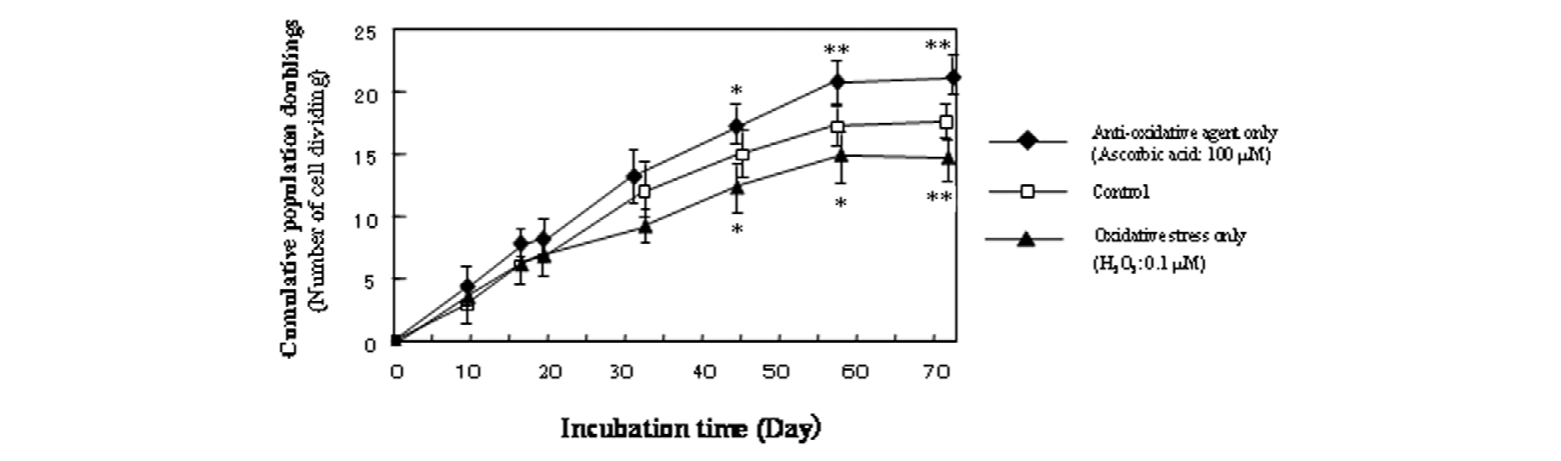
Chondrocyte replicative capacity under the various oxidative conditions. At each subculture (initial subculture at the start of the experiment: 5 × 10^4 ^cells/dish, primary culture), the total number of cells in the dish was determined, and the cells (1 × 10^5 ^cells/dish) were placed in a new dish. The number of cells that had attached 6 hours after seeding was determined. The increase in cumulative population doublings (number of cell divisions) at each subculture (*n *= 4 per treatment group) was calculated based on the number of cells attached and the cell yield at the time of the next subcultivation. Cell cultures were considered to have achieved their proliferative limit (senescence) when they did not exceed a twofold increase in 4 weeks. Values are expressed as mean ± standard deviation of four donors. *P < 0.05 and **P < 0.01, versus control group at each incubation time.

To clarify the effect of oxidative stress on the replicative potential of chondrocytes, we analyzed the cellular replicative potential of chondrocytes in the presence of the antioxidative agent or ROS *in vitro*. As shown in Fig. 3, the replicative potential of cultured chondrocytes was expressed as the cumulative number of cells dividing at each incubation time. After 20 days of incubation the H_2_O_2 _treated group exhibited lesser replicative potential as compared with the control group at any incubation time. In contrast, treatment with the antioxidative agent increased the cellular replicative potential at all incubation times after 20 days (Fig. 3).

During the 4 weeks after a 50- to 60-day incubation, the cumulative population doubling levels of all groups reached a plateau, indicating that the cultured chondrocytes in each group reached the limit of their ability to divide, namely cellular senescence, after about 8 weeks of incubation. The mean lifespan to cellular senescence was 23 population doublings in the antioxidative agent treated group, 18 population doublings in the control group, and 14 population doublings in the ROS-treated group (Fig. 3).

### Chondrocyte telomere length under the different oxidative conditions

**Figure 4:**
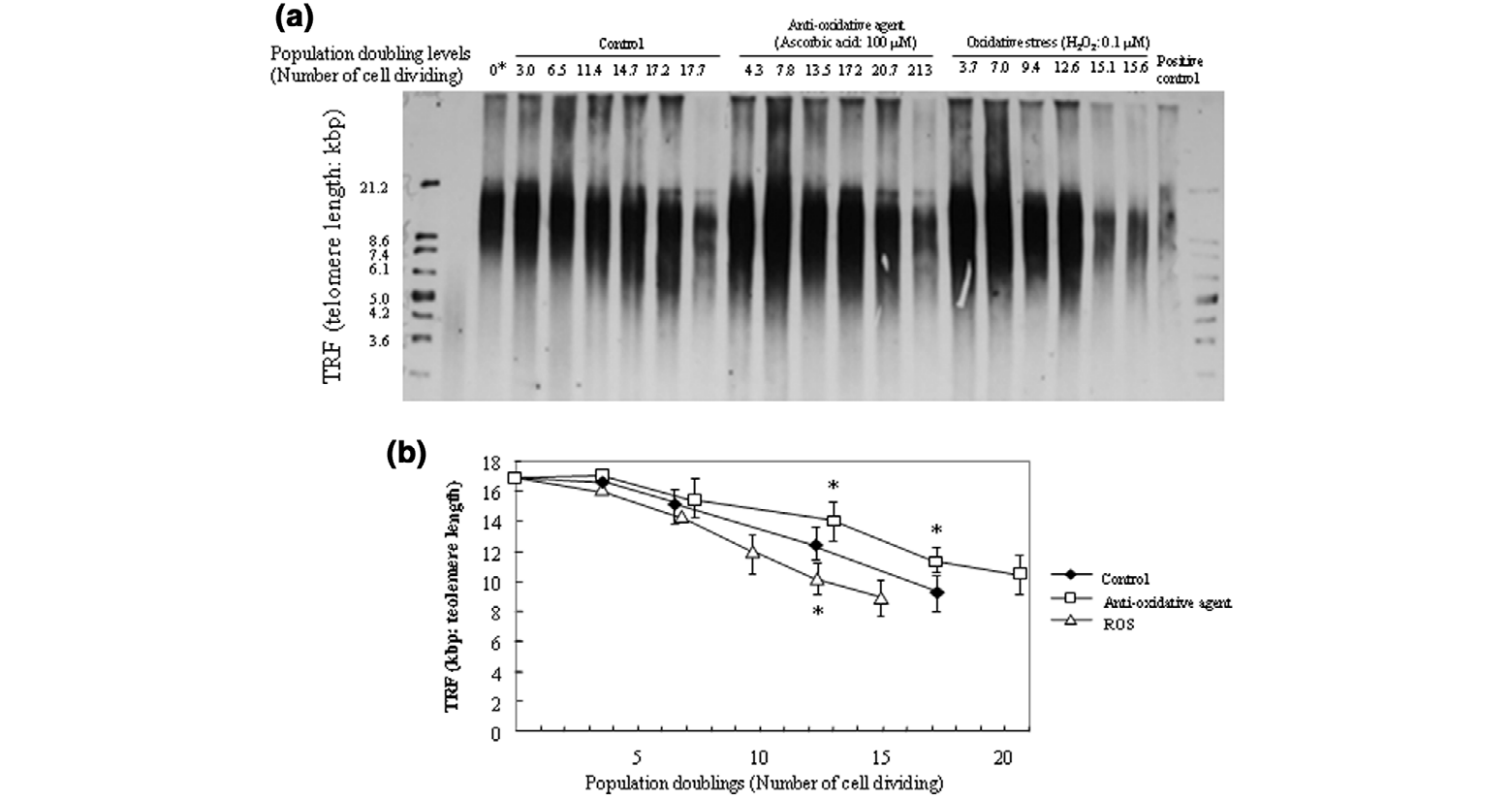
Southern blot analysis of chondrocyte telomere lengths in cultured chondrocytes at each passage under the different oxidative conditions. **(a) **Representative image of Southern blot analysis. Telomere lengths in chondrocytes (1 × 10^6 ^cells/dish, initial subculture at the start of the experiment: chondrocytes at passage 3 or 4) were determined using the terminal restriction fragment (TRF) assay. **(b) **The mean lengths of the chondrocytes were calculated by densitometric molecular weight analysis and were plotted against the number of cell population doublings. *P < 0.05, versus control group at each incubation time. ROS, reactive oxygen species.

To clarify the effect of oxidative stress on the telomeric instability in chondrocytes, we analyzed the telomere length of chondrocytes in the presence of an antioxidative agent or ROS *in vitro *(Fig. 4a). After five to six population doublings, telomere lengths of chondrocytes were shorter in H_2_O_2 _treated groups than in control groups at any level of population doubling. Treatment with an antioxidative agent resulted in a tendency of chondrocyte telomere length to elongate (*n *= 9; Fig. 4b).

### Immunohistochemical staining for nitrotyrosine of human articular cartilage cultured under different oxidative conditions

**Figure 5:**
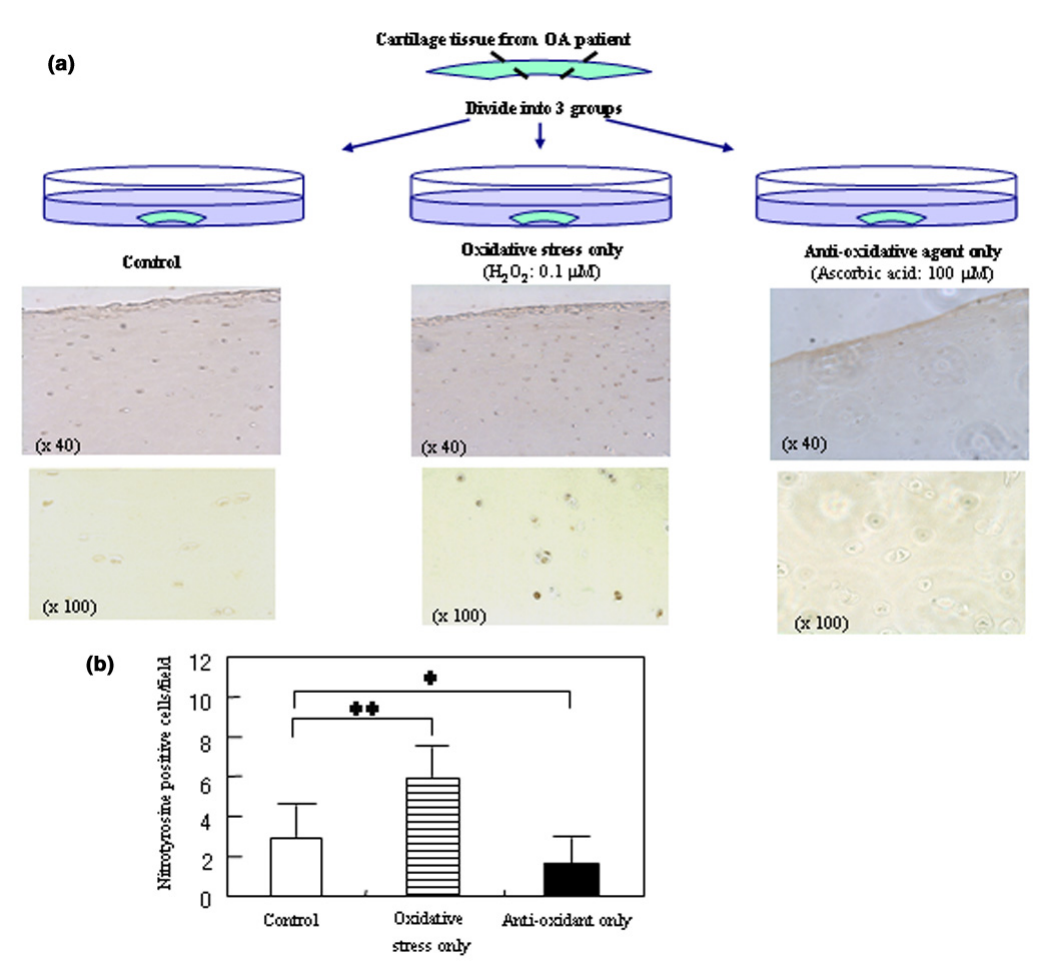
Tissue culture of articular cartilage tissue. **(a) **Representative immunohistochemical staining for nitrotyrosine in cartilage explants treated with reactive oxygen species (ROS) or an antioxidative agent in tissue culture. Osteoarthritis (OA) cartilage explant from a 67-year-old donor was cut and divided into three groups: control group, H_2_O_2 _treated group, and antioxidative agent (ascorbic acid-2-*O*-phosphate [Asc2P]) treated group. After the end of the incubation period (48 hours of incubation), the cartilage sections were immunostained with anti-nitrotyrosine antibody. Original magnifications are given in parentheses. **(b) **The number of nitrotyrosine positive cells were counted in the 20 areas of tissue-cultured cartilage at 200× magnification (0.785 mm^2^/field). A statistical analysis of immunostaining was performed. *P < 0.05, **P < 0.01, versus control group.

To examine the influence of an antioxidative agent or ROS in human articular cartilage, immunohistochemical staining for nitrotyrosine was evaluated in cartilage samples that were treated with an antioxidative agent or ROS (H_2_O_2_) in organ culture. Cartilage from an OA patient was cut and divided into three groups as follows: control group, antioxidative agent (Asc2P) treated group, and H_2_O_2 _treated group. After a 48-hour incubation in explant culture, OA articular cartilage in both the control group and the H_2_O_2 _treated group exhibited positive immunostaining for nitrotyrosine (Fig. 5a). The degree of nitrotyrosine staining was higher in the H_2_O_2 _treated group than in the control group (Fig. 5b). In contrast to these two groups, articular cartilage treated with the antioxidative agent showed less staining for nitrotyrosine (Fig. 5b).

### Catabolic changes to articular cartilage matrix under different oxidative conditions in organ culture

To investigate whether oxidative stress resulted in catabolic changes to the articular cartilage matrix, we examined the amount of GAG remaining in cartilage tissue and that was released into the culture medium in organ culture in the presence of an antioxidative agent or ROS. Catabolic changes to proteoglycan in the tissue were quantified as the percentage of proteoglycan remaining in the cartilage relative to total amount in the culture medium plus cartilage.

**Figure 6:**
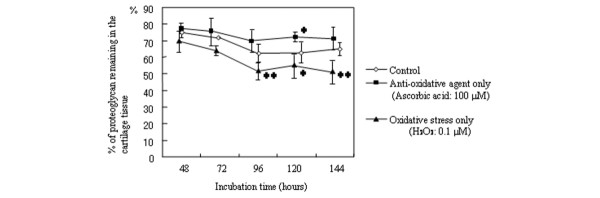
Glycosaminoglycan (GAG) remaining in the cartilage extract treated with reactive oxygen species (ROS) or antioxidative agent in tissue culture. Catabolic change in articular cartilage matrix was analyzed by determining the GAG content remaining in the cartilage extract relative to the total amount of GAG in the supernatant and the cartilage digest. Values are expressed as mean ± standard deviation of nine donors (three cartilage extracts per donor). *P < 0.05, **P < 0.01, versus control group at each incubation time.

During culture, the amount of proteoglycan remaining in the cartilage tissue in the control group and H_2_O_2_-treated group decreased gradually in a timedependent manner. After 72 hours of incubation, the percentage of proteoglycan remaining in the cartilage tissue was significantly lower in the H_2_O_2 _treated group than in the control group. In contrast, the antioxidative agent (Asc2P) treated group exhibited a tendency to maintain tissue proteoglycan even in the presence of H_2_O_2 _during the incubation period we studied in organ culture (Fig. 6).

### Telomere length of chondrocytes from human articular cartilage explants cultured under different oxidative conditions

**Figure 7:**
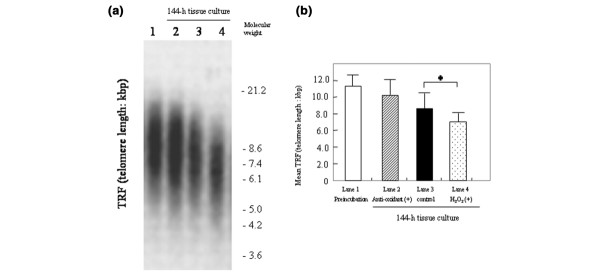
Telomere length of cultured chondrocytes from tissue cultured cartilage explants under the different oxidative conditions. After 144 hours' incubation of tissue culture, chondrocytes were isolated from cartilage explants, which were incubated in the presence or absence of H_2_O_2 _(0.1 μmol/l) or ascorbic acid-2-*O*-phosphate (Asc2P; 100.0 μmol/l). Telomere lengths in chondrocytes (1 × 10^6^chondrocytes of passage 3–4 after isolation) were determined using the terminal restriction fragment (TRF) assay. **(a) **Representative image of telomere length assay of chondrocytes after 144 hours of incubation. Lane 1, pretreated group (telomere length of isolated chondrocytes from cartilage explants before tissue culture); lane 2, Asc2P + H_2_O_2 _treated group; lane 3, control group; lane 4, H_2_O_2 _treated group. **(b) **Treatment with Asc2P (lane 2) showed a tendency to elongate the mean telomere length of chondrocytes in comparison with control. Mean telomere length in H_2_O_2 _treated group was significantly shorter than in the control group (*n *= 9; *P *< 0.05).

To clarify the effect of oxidative stress on chondrocyte telomeric instability in the cartilage, we analyzed the telomere length of chondrocytes that were isolated from cartilage explants cultured in the presence of an antioxidative agent (Asc2P) or ROS (H_2_O_2_) *in vitro*. After 144 hours of incubation, the telomere length of chondrocytes was significantly shorter in H_2_O_2 _treated groups (lane 4 in Fig. 7a,b) than in control group (lane 2 in Fig. 7b). Treatment with an antioxidative agent showed a tendency to maintain chondrocyte telomere length (lane 3 in Fig. 7).

## Discussion

The present study clearly demonstrates for the first time that oxidative stress affects chondrocyte telomeric DNA, cellular replicative lifespan, chondrocyte function, and cartilage matrix proteoglycan structure and composition *in vitro *and *in vivo*. These findings are consistent with a large body of data showing that reactive oxidative species, such as NO and ROS, are important in the pathogenesis of OA [11-16]. More recently, a suggestion that chondrocyte senescence may contribute to the risk for cartilage degeneration by decreasing the ability of the cells to maintain and to repair cartilage tissue has attracted attention [3-6]. Age-dependent changes in articular cartilage increase the risk for joint deterioration that causes the clinical syndrome of OA. However, the exact mechanism of chondrocyte senescence remains unclear. Our findings, demonstrating the oxidative stress (ROS) induced telomere erosion and replicative senescence in chondrocytes, suggest the involvement of oxidative stress in both the progression of cartilage ageing (chondrocyte senescence) and the development of OA.

Our results also show the presence of oxidative damage in degenerated cartilage from OA patients. Chondrocytes have been shown to be capable of producing ROS and NO [15,20,40]. In the present study, stronger staining for nitrotyrosine, a marker of oxidative stress, was observed in degenerating regions as compared with intact regions from the same articular cartilage samples. In addition, the degree of immunostaining was correlated with the level of histological change in articular cartilage. These findings suggest that local accumulation of proteins altered by the reaction between ROS and NO may be important in the pathogenesis of OA. Oxidative damage in cartilage may affect chondrocyte function, resulting in changes in cartilage homeostasis that are relevant to cartilage ageing and the development of OA.

We also measured the antioxidative potential of articular cartilage tissue using an assay based on reduction in Cu^2+ ^to Cu^+ ^by the combined action of all antioxidants present in the cartilage sample. Numerous reports have demonstrated that hypoxia is suitable for chondrocyte proliferation *in vitro *[41-43]. During chondrocyte differentiation, hypoxia may promote the process, although the exact mechanisms of chondrocyte differentiation have not been investigated to date. In addition, there is a general consensus that tissue oxygen partial pressures within articular cartilage decrease with increasing depth from the cartilage surface to deep layers [38,44,45]. Oxygen gradients do indeed exist in joint articular cartilage. These findings suggest that hypoxia may be required for homeostasis and maintenance of articular cartilage as well as chondrocyte cell growth and differentiation. During the development of OA, mechanical and chemical stresses may affect cellular adaptation to hypoxia, consequently leading to oxidative damage and changes in the microenvironment due to oxidative damage, resulting in the downregulation of chondrocyte synthesis. Indeed, our results revealed that antioxidative potential was significantly lower in degenerating regions than in intact regions from the same articular cartilage sample in OA.

To clarify the involvement of oxidative damage in the development of OA, we focused on chondrocyte telomere instability. Cumulative cell damage from oxidative stress provides an alternative explanation for cellular senescence. Oxygen free radicals directly damage guanine repeats in telomeric DNA, resulting in telomere erosion regardless of cell division [16-19]. DNA single strand damage by oxygen free radicals results in telomere shortening during DNA replication. Oxidative stress increases the telomere shortening rate by up to one order of magnitude [46]. From these findings, we postulated that oxidative stress directly induces chondrocyte telomere instability in OA cartilage tissue, resulting in chondrocyte senescence with no requirement for cell division. Our results, demonstrating chondrocyte telomere shortening in the presence of H_2_O_2_, at a noncytotoxic concentration, supports this hypothesis.

In addition to oxidative stress-induced telomere shortening, chondrocytes under chemical oxidative stress showed lower replicative lifespan and proteoglycan production as compared with normal chondrocytes *in vitro*. These findings also indicate that oxidative stress affects chondrocyte viability, and replicative potential and function, as well as telomere erosion.

We investigated catabolic changes to articular cartilage matrix under different oxidative conditions in tissue culture. The degree of immunostaining for nitrotyrosine was significantly higher in ROS (H_2_O_2_) treated cartilage tissues than in control cartilage tissues that were derived from the same articular cartilage. In addition, the GAG released to the medium was increased in the presence of ROS, suggesting that oxidative damage induces catabolic changes to cartilage matrix proteoglycan in articular cartilage. These observations led us to the hypothesis that oxidative stress may induce catabolic changes in cartilage matrix, consequently leading to the development of OA. This hypothesis is supported by the results of the present study, demonstrating that treatment of articular cartilage with the antioxidative agent ascorbic acid resulted in less immunopositivity for nitrotyrosine and maintenance of GAG content in articular cartilage in tissue culture.

Interestingly, treatment of cultured cartilage with an antioxidative agent not only inhibited GAG loss but also maintained telomere length of chondrocytes from cultured cartilage in contrast to data obtained from cultured cartilage under normal or ROS-treated conditions. These findings may very well indicate the role played by endogenous oxidative agents in catabolic changes to cartilage matrix proteoglycan and telomere length. This is an important observation and will validate the hypothesis that oxidative agents play a role *in situ *in chondrocytes and in cartilage changes in OA. These results also support the concept that antioxidative agents may prevent oxidative stress-induced chondrocyte dysfunction and degeneration in cartilage.

The findings of the present study suggest that cumulative oxidative stress leads to a decrease in antioxidative capacity in articular cartilage, resulting in chondrocyte telomere shortening, regardless of cell proliferation. Oxidative stress may be closely involved in telomere erosion, cellular senescence in chondrocytes and resultant cartilage ageing.

## Conclusion

This study provides insight into the involvement of oxidative stress in the pathogenesis of OA from the viewpoint of oxidative stress induced genomic instability, especially telomere erosion, and chondrocyte senescence. Our findings clearly show the presence of oxidative stress in degenerating cartilage, and the resultant telomere erosion and dysfunction of chondrocytes *in vitro *and *in vivo*, suggesting a role for oxidative stress in the development of OA. Also, our results suggest that antioxidative agents are effective in preventing and overcoming oxidative stress induced cartilage degeneration. New efforts to prevent the development and progression of OA may include strategies and interventions aimed at reducing oxidative damage in articular cartilage.

## Abbreviations

Asc2P = ascorbic acid-2-*O*-phosphate; DMEM = Dulbecco's modified Eagle's medium; GAG = glycosaminoglycan; NO = nitric oxide; OA = osteoarthritis; PBS = phosphate-buffered saline; ROS = reactive oxygen species.

## Competing interests

The author(s) declare that they have no competing interests.

## Authors' contributions

KY carried out *in vitro *studies (cell culture and organ culture), participated in the design of the study, conducted sequence alignment and drafted the manuscript. NvT carried out the immunoassays. HN, KH-M, TK and KN conceived the study, participated in its design and coordination, and helped to draft the manuscript. All authors read and approved the final manuscript
